# Impact of FODMAP Content Restrictions on the Quality of Diet for Patients with Celiac Disease on a Gluten-Free Diet

**DOI:** 10.3390/nu11092220

**Published:** 2019-09-14

**Authors:** Karla A. Bascuñán, Luca Elli, Nicoletta Pellegrini, Alice Scricciolo, Vincenza Lombardo, Luisa Doneda, Maurizio Vecchi, Cecilia Scarpa, Magdalena Araya, Leda Roncoroni

**Affiliations:** 1 Center for Prevention and Diagnosis of Celiac Disease, Gastroenterology and Endoscopy Unit. Fondazione IRCCS Ca’ Granda Ospedale Maggiore Policlinico, 20122 Milan, Italy; kbascunan@med.uchile.cl (K.A.B.); dottorlucaelli@gmail.com (L.E.); scricciolo.alice@gmail.com (A.S.); vicky.l@hotmail.it (V.L.); 2 Department of Nutrition, Medical School, University of Chile, 8380453 Santiago, Chile; 3 Human Nutrition Unit, Department of Food and Drug, University of Parma, 43124 Parma, Italy; nicoletta.pellegrini@unipr.it (N.P.); cecilia.scarpa@studenti.unipr.it (C.S.); 4 Department of Biomedical, Surgical and Dental Sciences, University of Milan, 20100 Milan, Italy; luisa.doneda@unimi.it; 5 Department of Pathophysiology and Transplantation, University of Milan, 20100 Milan, Italy; maurizio.vecchi@policlinico.mi.it; 6 General Surgery Unit, Fondazione IRCCS Ca’ Granda Ospedale Maggiore Policlinico, 20100 Milan, Italy; 7 Institute of Nutrition and Food Technology, INTA, University of Chile, 7830490 Santiago, Chile; maraya@inta.uchile.cl

**Keywords:** gluten-free diet, FODMAP, diet quality, nutritional adequacy, celiac disease

## Abstract

Restrictive diets as gluten-free (GFD) or reduced in Fermentable, Oligosaccharides, Disaccharides, Monosaccharides, and Polyols (FODMAP) are used to improve gastrointestinal (GI) symptoms in sensitive individuals. Aiming at comparing the nutritional quality and effects of a regular GFD regimen (R-GFD) and a low-FODMAP GFD (LF-GFD), in 46 celiac patients with persistent GI symptoms we conducted a randomized, double-blind intervention-controlled study. Patients received a personalized diet, either a strict GFD (*n* = 21) or a LF-GFD (*n* = 25) for 21 days. A validated food-frequency questionnaire before intervention and a 7-day weighed-food record after the intervention assessed the diets. Patients were 41.1 ± 10.1 years (mean ± SD), 94% women, with mean BMI 21.8 ± 2.9 kg/m^2^. On day 21, patients on R-GFD still showed poor nutritional adequacy compared to dietary recommendations, with decreased energy intake, even though an improvement in carbohydrates and folates was observed (all *p* < 0.025). In both groups, intake of iron, calcium, vitamin D, sodium and folates did not meet daily recommendations. As expected, consumption of legumes and grains was lower and that of fruits was higher in the LF-GFD group than in the R-GFD one (all *p* < 0.05). The nutritional quality of both diets was not different. When restrictive diets are useful to improve the persistent GI symptoms, careful nutritional surveillance and counseling is mandatory.

## 1. Introduction

The exclusion of dietary gluten is the only currently accepted treatment for gluten-related disorders [1], as Celiac Disease (CD). This is an autoimmune condition triggered by gluten, affecting mainly the small intestine in genetically susceptible individuals and exhibiting broad clinical manifestations [2]. The withdrawal of gluten from the diet implies the exclusion of all food containing wheat, rye, barley, spelta, and hybrids such as triticale. Although restrictive, the gluten-free diet (GFD) should be rich in nutrients with an adequate balance in macro- and micronutrients, including natural and processed gluten-free foods, easily accessible and at an affordable price [3]. Because the GFD that celiac patients maintain as treatment must be strict, permanent and maintained lifelong, it often results in a high burden on social life and health related quality of life [4,5], favoring poor compliance [6]. 

GFD is safe and effective. In most patients, it improves histological lesions, blood biochemistry, clinical manifestations and decreases the risk of complications [7]. However, some patients do not show complete clinical remission despite following strict GFD; these patients report persistent gastrointestinal symptomatology resembling Irritable Bowel Syndrome (IBS) [8]. Studies restricting fermentable oligosaccharides, disaccharides, monosaccharides, and polyols (FODMAP) intake has proved efficacious for IBS management [9]. FODMAP are poorly absorbed short-chain carbohydrates, including fructose, lactose, polyols, fructans, and galacto-oligosaccharides [10]. We were first to report potential benefits of FODMAP restriction in celiac patients on GFD and with persisting functional gastrointestinal symptoms [11]. We showed that a short-term low-FODMAP diet improves gastrointestinal symptomatology and psychological health, enhancing patients’ well-being. Another study has reported consistent results when evaluating the combination of both diets in the treatment of Non-Celiac Gluten Sensitivity (NCGS), showing significant clinical and psychological symptom improvements in these patients [12].

Both GFD and low-FODMAP diets are characterized by an important restriction of food categories (i.e., grains in GFD and plant-based foods in low-FODMAP diet (LFD) and applying them together may have harmful nutritional consequences. Calcium and short-chain carbohydrates intake has been reported to be reduced in patients on a low-FODMAP diet [13]; however, a recent study showed that nutritional adequacy was not deteriorated in patients following a low-FODMAP diet even after a long time (18 months) [14]. On the other hand, higher fat, sugar, and energy content is often reported in the diet of CD patients, as a consequence of the gluten-free foods composition [15]. A lower intake of micronutrients such as magnesium, iron, zinc, manganese, and folate have also been reported in CD patients [3,16,17]. Aiming at improving our knowledge in this area, in this present study, we aimed at comparing the nutritional quality of the regular GFD (R-GFD) and a short-term low-FODMAP GFD (LF-GFD) regimen, in celiac patients already on GFD.

## 2. Materials and Methods 

This study involved CD patients participating in a randomized, double-blind intervention-controlled study (previously registered at ClinicalTrials.gov with ref. no. IDNCT02946827), which assessed the effect of a GFD combined with a LFD on GI symptoms, as previously described [11]. Patients were 41.1 ± 10.1 (mean ± SD) years of age, mainly women (94%) with a mean body-mass index of 21.8 ± 2.9 kg/m^2^. Inclusion criteria were: adults (18 to 60 years old), treated with GFD for at least a year, with negative plasma tissue transglutaminase values and IBS-like symptoms (functional gastrointestinal disorders according to the Rome III criteria) [18], with a global well-being score < 4 assessed by a visual analogue scale. Exclusion criteria were: low adherence to GFD as evaluated by the Celiac Dietary Adherence Test [19]; refractory CD, as evaluated by i) small intestinal biopsy to assessed the persistence of intestinal atrophy while on GFD and ii) an interview by a trained nutritionist, who assessed patients’ adherence to the diet; individual intolerance to disaccharides lactose and fructose as evaluated by hydrogen test; a history of previous nutritionist evaluation or nutritional treatment for IBS dietary management; IBS pharmacological therapy; abdominal surgery; and type-2 diabetes. CD was diagnosed by positive serological tests -anti endomysium antibodies and anti-tissue transglutaminase antibodies- and duodenal histological abnormalities that followed the modified Marsh classification (following the American College of Gastroenterology clinical guidelines) [20]. The patients were recruited at the Center for Prevention and Diagnosis of Celiac Disease of Fondazione IRCCS Ca’ Granda Ospedale Maggiore Policlinico in Milan. The Institutional Review Board of the University of Milan reviewed and approved the study protocol (Project Identification Code 744_2015bis). All patients fulfilling the inclusion criteria and agreeing to participate were enrolled. A signed written informed consent was obtained from all patients prior to incorporation to the protocol.

### 2.1. Intervention Diets

A personalized GFD adjusted to match the daily requirements of energy, macronutrients, and micronutrients was calculated for each patient by a trained nutritionist, who was not involved in the patients’ management. In each case, an in-depth GFD review and food education regarding GFD and LFD (in the LF-GFD group) was provided to the patient; then a structured 21-day dietary plan was given to the participants, which excluded all sources of dietary gluten. This plan included daily meals and specific foods/beverages. After the initial explanation, the nutritionist addressed doubts related to the dietary plan via e-mail or telephone thereafter. Changes in FODMAP dietary content included dietary counseling on how to start changing FODMAP consumption towards LFD. The FODMAP content of the R-GFD and LF-GFD included a median amount (interquartile range) of 21.8 (18.5–22.5) and 3.7 (3.0–4.12) g/day, respectively, as previously described [11,21,22]. Compliance to and doubts on the dietary plan were assessed ten days later by telephone call or e-mail by a nutritionist. On day 14, patients were instructed to record their daily consumption in a 7-day weighed food diary and return it completed on day 21. At this time, a second nutritional interview was carried out by the same nutritionist that assessed the dietary data during the intervention period. 

### 2.2. Nutritional Assessment

Diets’ characteristics were assessed at twice: before the intervention started (through a validated food-frequency questionnaire (FFQ)) and at the end of the intervention period (by means of the 7-day weighed food diary where the dietary information was recorded between day 14 and 21). The FFQ was administered by a trained nutritionist, who obtained information about food consumption during the previous year. 

### 2.3. Dietary Evaluation at Baseline

The electronic version of the EPIC FFQ, developed for northern-central Italy and specifically adapted for the celiac population (including 188 food items), was used to establish the usual intake of food and beverages consumed during the year prior to this study [17]. In the questionnaire, each respondent was asked to indicate the number of times any given food/beverage was consumed (per day, week, month, or year). Participants selected an image of a food portion (a pre-defined standard portion was used when no image was available) to quantify the portion size. This instrument does not ask about the frequency of intake and dosages of commonly consumed dietary supplements. The nutritional composition of food items listed in the EPIC FFQ was modified as described previously [17] to include the recipes of composite gluten-free foods and generic gluten-free commercial foods. Complex foods were split into their ingredients, and the gluten-free products with the closest ingredient composition was used. In doing so, definition of an appropriate alternative of gluten-free food was based mainly on energy and carbohydrates composition. For the modified EPIC FFQ 24 foods containing gluten were replaced with 24 gluten-free foods. An ad-hoc computer program (Nutritional Analysis of Food Frequency Questionnaire) developed by the Epidemiology and Prevention Unit of the IRCCS Foundation, National Cancer Institute of Milan, was used to convert the questionnaire’s dietary data into the frequencies of consumption and mean daily quantities of foods (grams per day), energy, and nutrients consumed. The food items contained in the FFQ were grouped into the same food groups identified for the 7-day weighed food diary, based on the similarities in the nutrient profile and culinary usage.

### 2.4. Dietary Evaluation at the End of Intervention 

The total food and beverage consumption was assessed using the 7-day food diary, filled on days 14–21 [23]. At baseline and on the day 14 visit, the participants were instructed by a nutritionist on how to record all foods consumed and the dairies were reviewed by the nutritionist with the patient on day 21 to clarify doubts. These food diaries were sent to the Department of Food and Drug of the University of Parma for processing. Nutrient intake was calculated by means of a Microsoft Access application (version 2003, Microsoft Corp., Redmond, WA, USA) linked to the European Institute of Oncology’s food database, which covered the nutrient composition of 900+ Italian foods [24], integrated with the nutrient composition of 60 gluten-free foods available in the Italian market [25]. When a food recorded by the participant was not be found in the database, an alternative food was appropriately chosen based on its similarities in energy and nutrient composition. The output consisted of the daily intake of energy and nutrients for each patient. The food items of interest for this study were grouped into the following categories: pasta, bread (including crackers and salted snacks), cereals (including corn, quinoa, buckwheat, and rice), fruits, vegetables, legumes, potatoes, sweeteners (honey, saccharin, fructose, barley malt syrup) and sweets (including biscuits, sweet snacks, breakfast cereals, ice-cream, candies, and chocolate), dried fruits, lipids (oil and fats), dairy products (including milk, yogurt, cream, cheese), eggs, fish meats, soft drinks, juices, coffee/tea, and alcoholic beverages. For each patient, the mean daily intake of each food category was calculated. Nutrient, adequacy was calculated against the Recommended Dietary Allowances (RDA). For each nutrient, adequacy was considered if the calculated nutrient intake was at least equal or higher than the respective daily RDA for that nutrient, according to the Institute of Medicine, National Academies, USA [26]. The adequacy of the energy intake was calculated as the energy intake relative to the estimated energy expenditure, (i.e., (energy intake/energy expenditure) × 100).

### 2.5. Statistical Analysis

Data were described as median ± Standard Deviation (SD) or median (inter-quartile range), depending on the parametric or non-parametric distribution of variables. The data distribution was assessed by graphical inspection and the Shapiro–Wilk test. The *Χ*^2^-test or Fisher’s exact two-tailed test were used for nutrient adequacy comparison between the baseline and last-week intervention within groups. The independent sample Student’s *t*-test was used to compare nutritional intake and adequacy of critical nutrients between groups at the last week of intervention. The non-parametric Wilcoxon rank-sum test was used to evaluate differences regarding the food groups consumption at the last week of intervention. A 5% significance level was used, and the software packages STATA^®^ v. 13.1 (StataCorp LLC, College Station, TX, USA) and GraphPad Prism v. 6 (GraphPad Software, La Jolla, CA, USA) were used for analysis and figures processing.

## 3. Results

### Nutritional Adequacy of Consumed Diets Compared to Macro- and Micronutrients Recommendations

Nutritional composition and adequacy to daily nutrient recommendations of 46 celiac disease patients GFD (n = 21) or a LF-GFD (n = 25) were analyzed, in Table 1 both groups are shown. In the R-GFD group, at baseline there was excess energy intake and poor compliance of carbohydrates (9/21 subjects) and fat (7/21 subjects) recommendations. For micronutrients, the lowest degree of adequacy was observed for vitamin D, folate, calcium, iron, sodium and potassium (Table 1). Changes in diet sufficiency during the last week of intervention were evaluated, revealing a significant decrease in energy adequacy (*p* = 0.0001) and an improvement in the adequacy of carbohydrates (*p* = 0.025). Regarding micronutrients, the only significant difference found was in folates, with better achievement of daily recommendations at the end of the intervention (*p* = 0.009, Table 1).

At baseline, the same analysis in the LF-GFD group showed adequate energy and protein sufficiency but poor compliance to carbohydrates and fat recommendations. In both groups, a low proportion of patients complied to the micronutrient adequacy of vitamin D, folates, vitamin E, iron, sodium, potassium and calcium. Dietary adequacy changed in the last week of intervention with lower energy adequacy (*p* = 0.048) and improvement in carbohydrates and fat adequacy (both *p* = 0.0001); there were no changes in the adequacy of evaluated micronutrients in the last week of intervention (Table 1).

**Figure 1:**
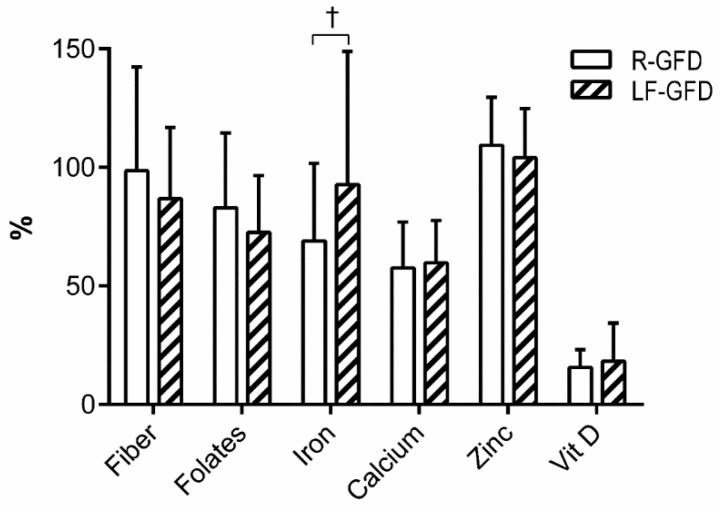
Adequacy level of critical nutrients between groups against the dietary recommendations. Data as mean ± SD. ^†^ Adequacy: [nutrient intake/nutrient daily recommendation (RDA)] × 100. Independent samples *t*-test: † *p* = 0.081. R-GFD: Regular gluten-free diet, LF-GFD: Low-FODMAP gluten-free diet.

At the end of the intervention period, comparison of the nutritional intake composition between the two groups showed similar daily intake of macronutrients and micronutrients, except for higher intake of animal protein (*p* = 0.037), cholesterol (*p* = 0.011), and vitamin C (*p* = 0.033) in the LF-GFD group than in R-GFD group (Table 2). The level of adequacy of critical nutrients folates, iron, calcium, and vitamin D intake was below the daily recommendations in both groups (Figure 1), with iron adequacy tending to increase in the LF-GFD group over the intervention period (*p* = 0.081).

**Figure 2:**
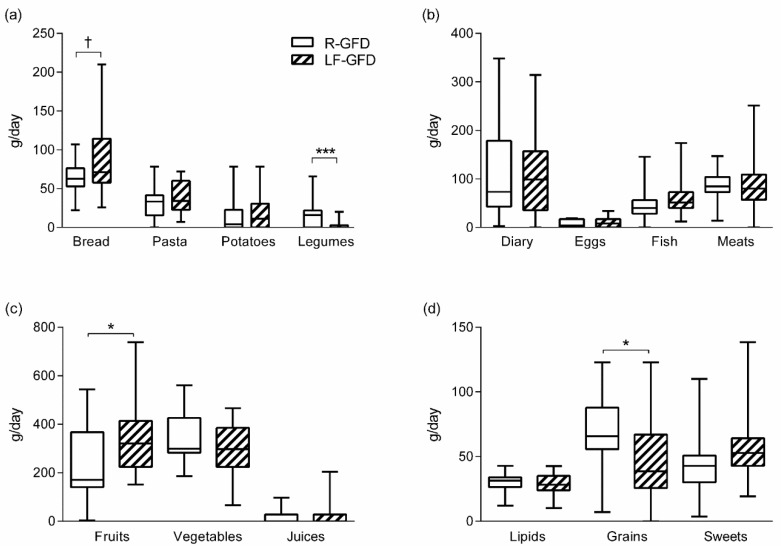
Food categories consumption between the groups at the end of intervention. Data as median. (horizontal line), interquartile range (p25‒p75, box), and minimum and maximum (whiskers). (**a**–**d**) show to different food classifications. Wilcoxon’s Rank Sum Test: * *p* < 0.05; *** *p* = 0.0008; ^†^ *p* = 0.098. R-GFD: Regular gluten-free diet; LF-GFD: Low-FODMAP gluten-free diet.

At the end of the intervention period, evaluation of relevant food groups was performed by quantifying the consumption of relevant food groups (Figure 2). Overall, comparison of most food groups showed no differences between diets; especially, dairy products, eggs and meats consumption did not differ between groups However, in the LF-GFD group there was a trend to have higher bread consumption whereas legumes consumption was significantly lower (*p* = 0.008) and consumption of fruits was higher and grains lower than R-GFD (both *p* < 0.05, Figure 2).

## 4. Discussion

In this study, we evaluated celiac patients with persistent gastrointestinal symptoms that followed either R-GFD or a diet that additionally restricted FODMAPs content. Nutrient adequacy in patients on R-GFD was poor when compared to dietary recommendations and it did not improve when a low-FODMAP diet was implemented. When comparing both types of diet at the end of the intervention period, slight differences were detected with regard to intake of animal protein, cholesterol, and vitamin C. When restricting FODMAP content, food groups consumption showed the expected changes, mainly a lower intake of legumes and grains and a higher fruit consumption compared to the R-GFD group. As a whole, our results show low nutritional quality of the GFD regimen and that the exclusion of FODMAP-rich foods from the diet does not worsen its nutritional quality. Both diets can be used as an alternative treatment for selected patients who continue with persistent symptomatology when following GFD.

The adherence to GFD by CD patients was considered to be nutritionally adequate when retrospective evaluated along the years [27]. Currently, several reports point out that patients on GFD should be continuously monitored to detect and prevent nutritional deficiencies that may develop in some individuals as well as affecting the practice of GFD [28,29,30]. It is widely agreed that the adverse nutritional impact of CD is related to the duration of the untreated state of the disease, the extension, and location of the mucosal lesions, and the degree of malabsorption of specific nutrients [31]. In this study, we evaluated the intake of some critical nutrients as proposed by others [3], showing that nutritional adequacy was achieved for zinc and fiber while was not for iron, folates, calcium, vitamin D, potassium and sodium. It is worth noting that nutritional supplements consumption was not included in our dietary analysis. We also observed that the group receiving R-GFD regimen showed some differences at the end of our intervention, as compared with their baseline assessment. After the overall evaluation and reinforcement of GFD on day 14, the diet quality improved as they reduced the energy intake and improved compliance to carbohydrates recommendations, a relevant issue considering the current scenario of overweight/obesity observed in several celiac patients [32].

The importance of nutritional quality of GFD has been more and more emphasized over the last decades. One study [33] showed an increased BMI after GFD initiation together with a higher (almost doubled) percentage of overweight subjects while they were on GFD. The authors speculated that this may be a consequence of incorrect eating habits, influenced by expensive commercial gluten-free products with poor nutritional quality and high-fat content [33]. In this study, post-intervention improvements (after 21 days on R-GFD) showed that folates intake also increased, suggesting that follow-up and reinforcement of dietary indications are essential to improve macro- and micronutrients intake. Therefore, a patient’s periodic monitoring with a trained nutritionist for prescribing and guiding GFD is of paramount importance [3].

FODMAP dietary restriction improved the persistent symptoms in our celiac patients that were already on GFD [11]. The physiological principle that supports FODMAP restriction is based on the fact that the incomplete hydrolysis/absorption short-chain carbohydrates in the small intestine reach the colon and are fermented by the microbiota, generating increased intestinal water and colonic gas [34]. However, it has been reported that FODMAP exclusion may lead to nutritional inadequacy celiac disease [35]. 

The resulting big question then is whether restriction of FODMAP in patients that are already on GFD may deteriorate nutrients intake. Results of this study show that this was not the case; after implementing the low-FODMAP diet there were no significant differences in energy or macronutrients intake as compared with R-GFD. Instead, there was an increase in animal protein, cholesterol, and vitamin C at the end of the intervention period as compared with R-GFD. However, comparing the energy and macronutrients intake pre- and post-intervention in the LF-GFD group, there was a significant decrease in the energy intake, a change that did not differ from the behavior of the R-GFD group. This issue has already been described in previous studies that report a lower carbohydrate and energy intake in subjects following a low-FODMAP diet as compared with their pre-intervention usual diet [36,37]. However, it has also been shown that the energy and macronutrient intake after a low-FODMAP diet was not different from their usual control diet [38].

Although GFD has some nutritional deficiencies, in this study we only found a trend for better adequacy of the iron intake in LF-GFD in comparison with the patients on R-GFD.

The analysis of the food groups shows the expected results regarding the food restriction in a low-FODMAP diet. However, such a restriction of specific foods is subject to the re-challenge phase. As part of the re-challenge phase-specific dietary triggers must be identified, and well-tolerated foods are re-introduced. The role of a nutritionist is crucial to assist patients in identifying specific dietary triggers, in reducing the level of dietary restriction, and increasing the prebiotic intake [39]. The re-challenge supports the re-introduction of a greater variety of foods by making food choices more flexible, arriving at one’s personal version of a modified LFD [40]. Our results also emphasize that these patients should be managed by a specialized nutritionist, who should educate, control and follow the restrictive diets (R-GFD and LF-GFD), ensuring that nutritional adequacy is reached and maintained [4] and that the impact on patients’ quality of life due to the restrictive diet is as low as possible.

Our study has some limitations that we would like to mention. First, we studied a relatively small sample of patients with CD and both dietary treatments were conducted during a rather short period of time. Further studies should evaluate the effect of FODMAP restriction in CD patients during a longer term. Second, the use of a structured 21-day diet plan that included daily meals and specific foods/beverages, which was designed individually for each patient, could clearly impact the composition of the diets as opposed to what patients might select them when instructed to follow the basic rules of such diets. Therefore, the findings may not reflect real-world practice where patients choose their own meals within the confines of the instructed diet. However, this allowed us ensuring that patients effectively consumed foods according to planned quantity and quality, and better compliance with the designed diets. Third, we used different methods for the assessment of food intake (FFQ and 7-day food diary) in our patients. This differential approach was chosen as the use of FFQ allowed us to evaluate patients’ usual dietary pattern while the 7-day food diary evaluated food intake during the last week of the intervention period, allowing us to acutely evaluate food intake under both diets.

## 5. Conclusions

This study demonstrates that patients with CD fail to meet relevant nutritional recommendations and shows an overall low diet quality. A three-week low-FODMAP diet/GFD, when applied by a specialized nutritionist, does not significantly impact on nutrient intake as compared with a regular GFD regimen and helps mitigating persistent gastrointestinal symptoms. However, this study applied the two diets and evaluated their effect only for 21 days. Further studies are necessary to confirm our results in long-term studies. When a low-FODMAP diet is prescribed to celiac patients on GFD, they must be supervised and periodically assessed by a specialized nutritionist. This will help improving the patients’ nutritional state and his/her quality of life.

## Figures and Tables

**Table 1:** Nutritional composition of diets and nutritional adequacy of macro and micronutrients against the daily recommendations in both groups.

	R-GFD Group				LF-GFD GRoup					
	Baseline (*n* = 21)	Adequacy ^†^	End of Intervention (*n* = 21)	Adequacy ^†^	Baseline (*n* = 25)	Adequacy ^†^	End of Intervention (*n* = 25)	Adequacy ^†^	*p* Value R-GFD ^‡^	*p* Value LF-GFD ^‡^
Energy, kcal ^††^	2212.4 ± 511.7	111.9 ± 30.0	1556.2 ± 220.4	78.8 ± 14.3	1837.0 ± 481.5	91.5 ± 27.8	1578.0 ± 238.9	78.9 ± 17.6	**0.0001**	**0.048**
Protein, %	13.8 ± 2.6	19 (90.4)	15.6 ± 2.2	21 (100.0)	16.2 ± 2.7	25 (100.0)	16.8 ± 3.3	24 (96.0)	0.147	0.999
Carbohydrate, %	44.4 ± 8.2	9 (42.8)	49.3 ± 4.5	17 (80.9)	41.2 ± 7.1	5 (20.0)	50.4 ± 3.8	23 (92.0)	**0.025**	**0.0001**
Fat, %	39.4 ± 6.3	7 (33.3)	35.7 ± 4.1	8 (38.0)	42.4 ± 6.2	2 (8.0)	33.9 ± 3.8	18 (72.0)	0.747	**0.0001**
Dietary fiber, g	26.0 ± 8.4	13 (61.9)	24.2 ± 10.8	7 (33.3)	21.0 ± 5.7	10 (40)	21.9 ± 8.8	8 (32.0)	0.064	0.556
Thiamin, mg	0.9 ± 0.2	8 (38.0)	0.9 ± 0.2	5 (23.8)	0.9 ± 0.2	5 (20.0)	0.9 ± 0.2	6 (24.0)	0.317	0.733
Riboflavin, mg	1.5 ± 0.4	19 (90.4)	1.5 ± 0.4	17 (80.9)	1.6 ± 0.6	21 (84.0)	1.4 ± 0.3	23 (92.0)	0.663	0.667
Niacin, mg	19.5 ± 3.8	20 (95.2)	19.6 ± 4.7	19 (90.4)	20.0 ± 6.7	20 (80.0)	20.6 ± 6.8	20 (80.0)	0.990	0.990
Vitamin B6, mg	2.0 ± 0.4	21 (100.0)	1.9 ± 0.4	21 (100.0)	2.1 ± 0.7	24 (96.0)	1.9 ± 0.3	25 (100.0)	-	0.990
Vitamin C, mg	120.5 ± 46.5	17 (80.9)	146.0 ± 82.7	19 (90.4)	138.9 ± 92.2	22 (88.0)	207.4 ± 107.0	24 (96.0)	0.663	0.609
Vitamin E, mg	12.7 ± 4.1	9 (42.8)	14.0 ± 2.7	7 (33.3)	11.8 ± 4.0	5 (20.0)	13.6 ± 2.3	7 (28.0)	0.525	0.508
Vitamin D, g	2.9 ± 1.1	0 (0)	2.3 ± 1.1	0 (0)	3.5 ± 3.0	1 (4.0)	2.7 ± 2.4	0 (0)	-	0.990
Folate, g	261.2 ± 68.6	0 (0)	331.8 ± 126.7	7 (33.3)	274.1 ± 89.1	1 (4.0)	290.3 ± 96.0	4 (16.0)	**0.009**	0.349
Calcium, mg	804.6 ± 391.3	3 (14.2)	599.7 ± 198.8	0 (0)	879.8 ± 434.8	7 (28.0)	601.2 ± 170.5	2 (8.0)	0.072	0.066
Iron, mg	11.0 ± 3.0	3 (14.2)	10.8 ± 3.6	2 (9.5)	10.4 ± 3.0	5 (20.0)	11.0 ± 3.2	9 (36.0)	0.990	0.208
Phosphorus, mg	1244.9 ± 374.6	20 (95.2)	1002.4 ± 209.5	18 (85.7)	1239.7 ± 454.9	24 (96.0)	1019.2 ± 205.9	24 (96.0)	0.606	0.990
Sodium, mg	2455.8 ± 846.0	2 (9.5)	4056.5 ± 1146.5	2 (9.5)	1861.8 ± 540.6	4 (16.0)	4243.5 ± 1374.5	3 (12.0)	0.990	0.684
Potassium, mg	3193.8 ± 697.4	0 (0)	2937.1 ± 750.9	0 (0)	3122.7 ± 957.6	1 (4.0)	3186.7 ± 772.7	2 (8.0)	-	0.990
Zinc, mg	9.7 ± 2.3	17 (80.9)	8.8 ± 1.7	15 (71.4)	9.5 ± 3.3	17 (68.0)	8.6 ± 1.7	14 (56.0)	0.719	0.382

Data are expressed as mean ± SD for the absolute intake of nutrients, and as frequency and (percentage) for their adequacy level; ^†^ For each nutrient, adequacy was achieved if the calculated nutrient intake was at least equal or higher than the respective nutrient daily Recommended Dietary Allowance (RDA), according to the Institute of Medicine, National Academies, USA. [26]; ^††^ The energy adequacy was calculated as the energy intake relative to the estimated energy expenditure: (energy intake/energy expenditure) × 100. For protein, carbohydrates, and fat adequacy, RDA is 10%–35%, 45%‒65%, and 20%‒35% of the energy intake. ^‡^ For comparison of nutrients adequacy between baseline and the end of intervention, a chi-square or Fisher’s exact test was used. Numbers in bold highlight significant differences between groups. R-GFD: Regular gluten-free diet; LF-GFD: low-FODMAP gluten-free diet.

**Table 2:** Comparison of nutritional daily intakes between the R-GFD and LF-GFD groups at the end of intervention ^1^.

	R-GFD (*n* = 21)	LF-GFD (*n* = 25)	*p* Value
Energy, kcal	1556.2 ± 220.4	1578.0 ± 238.9	0.750
Energy adequacy, %	78.8 ± 14.3	79.0 ± 20.2	0.959
Total protein, g	59.6 ± 10.9	65.7 ± 17.3	0.154
Protein, % of energy	15.6 ± 2.2	16.8 ± 3.3	0.133
Animal protein, g	36.3 ± 9.4	44.2 ± 15.4	0.037
Vegetal protein, g	23.1 ± 7.0	21.2 ± 5.1	0.311
Total carbohydrate, g	190.9 ± 31.4	198.4 ± 32.7	0.431
Carbohydrate, % of energy	49.3 ± 4.5	50.4 ± 3.8	0.376
Total fat, g	62.1 ± 11.7	59.6 ± 11.0	0.463
Fat, % of energy	35.7 ± 4.1	33.9 ± 3.8	0.123
Cholesterol, mg	148.3 ± 35.0	178.4 ± 42.6	0.011
Dietary fiber, g	24.2 ± 10.8	21.9 ± 8.8	0.433
Thiamin, mg	0.9 ± 0.2	0.9 ± 0.2	0.820
Riboflavin, mg	1.5 ± 0.4	1.4 ± 0.3	0.741
Niacin, mg	19.6 ± 4.7	20.6 ± 6.8	0.573
Vitamin B6, mg	1.9 ± 0.4	1.9 ± 0.3	0.637
Vitamin C, mg	146.0 ± 82.7	207.4 ± 107.0	0.033
Vitamin E, mg	14.0 ± 2.7	13.6 ± 2.3	0.642
Vitamin D, g	2.3 ± 1.1	2.7 ± 2.4	0.477
Folate, g	331.8 ± 126.7	290.3 ± 96.0	0.225
Calcium, mg	599.7 ± 198.8	601.2 ± 170.5	0.978
Iron, mg	10.8 ± 3.6	11.0 ± 3.2	0.874
Phosphorus, mg	1002.4 ± 209.5	1019.2 ± 205.9	0.786
Sodium, mg	4056.5 ± 1146.5	4243.5 ± 1374.5	0.617
Potassium, mg	2937.1 ± 750.9	3186.7 ± 772.7	0.274
Zinc, mg	8.8 ± 1.7	8.6 ± 1.7	0.695

^1^ Data are shown as mean ± SD. *p*-value for comparison between the groups using independent samples *t*-test. R-GFD: Regular gluten-free diet, LF-GFD: Low-FODMAP gluten-free diet.
